# Supplementary material for: Genetic correlates of phenotypic heterogeneity in autism
Source: Nat Genet. 2022 Jun 2;54(9):1293–304. doi: 10.1038/s41588-022-01072-5 (PMC9470531; doi:10.1038/s41588-022-01072-5)
Supplement: Supplementary file 1 — Supplementary Figs. 1–9. [file 41588_2022_1072_MOESM1_ESM.pdf]

nature portfolio

https://doi.org/10.1038/s41588-022-01072-5

Supplementary information

Genetic correlates of phenotypic heterogeneity in autism

In the format provided by the authors and unedited

Supplementary Figure 1: Flowchart of the factor models tested

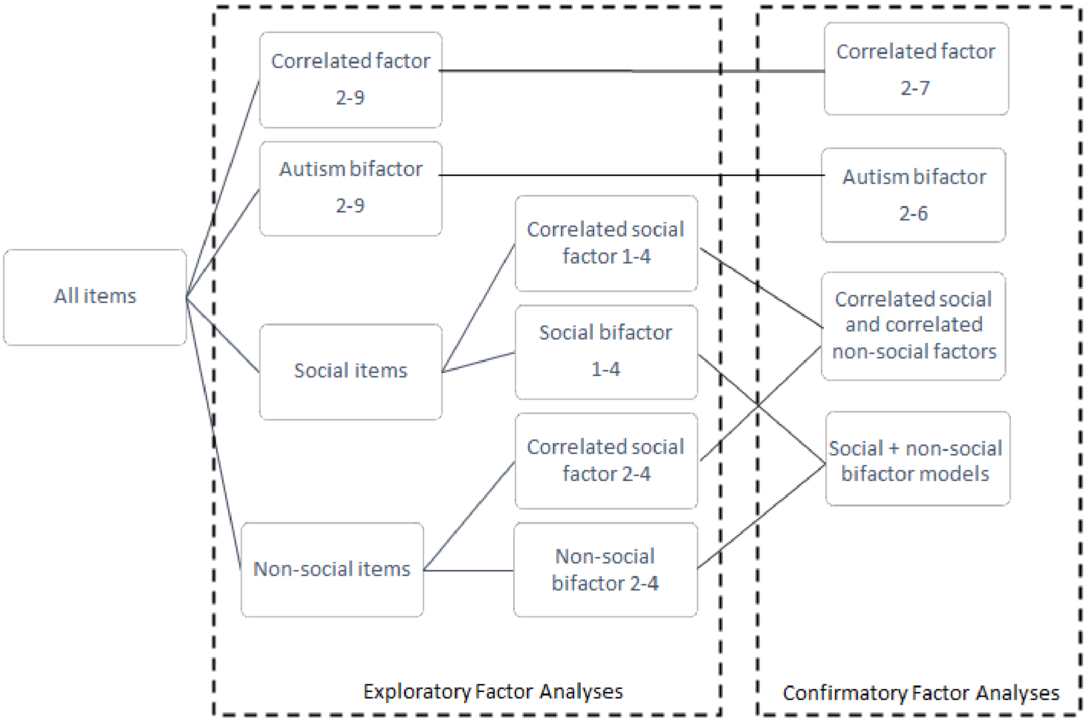

We tested 42 models in total using a series of exploratory and confirmatory factor analyses. This includes two sets of correlated factor models (correlated factor, and correlated social and correlated non-social factors), and two sets of bifactor models (Autism bifactor and social + non-social bifactor models).

Supplementary Figure 2: Scree plots for the exploratory factor models

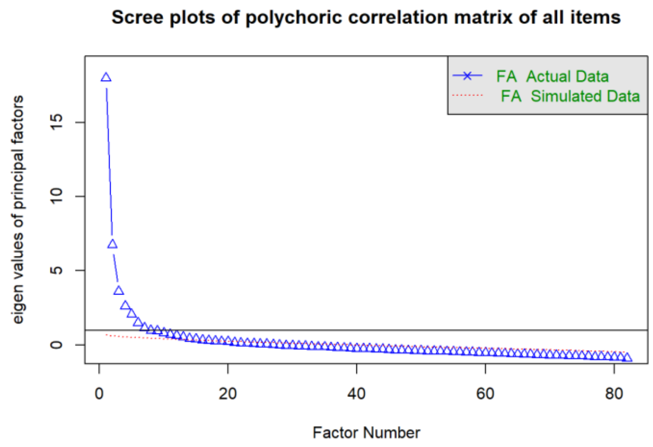

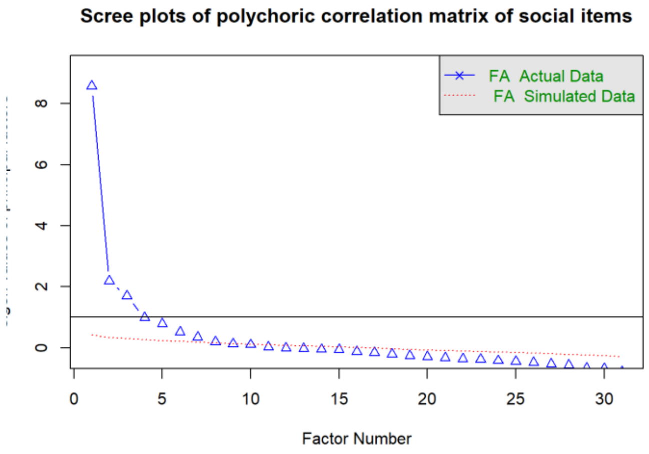

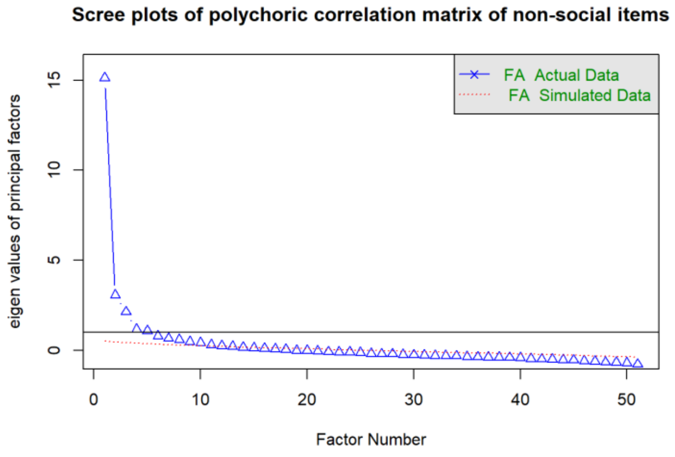

Scree plots for: (1) Correlated factor; (2) Correlated social factor; and (3) Correlated non-social factor models. Examination of the scree plot suggested 6 correlated factors, 2 social factors and 4 non-social factors.

Supplementary Figure 3: Factor scores by sex and full-scale IQ bins

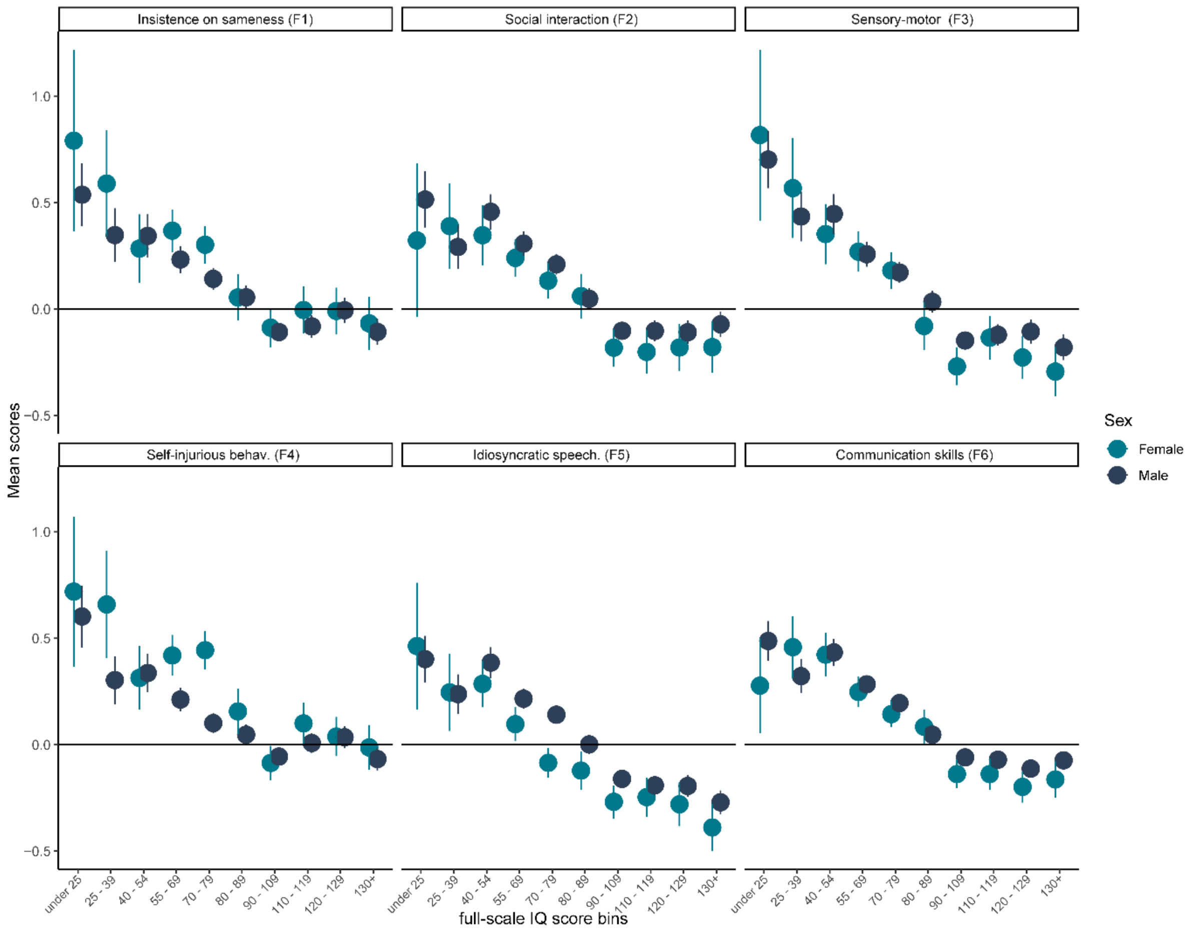

Mean scores and 95% confidence intervals for the six factor scores in 10 full-scale IQ bins, stratified by sex. N = 8,899 males and 2,472 females.

Supplementary Figure 4: Age related trajectories in factor scores

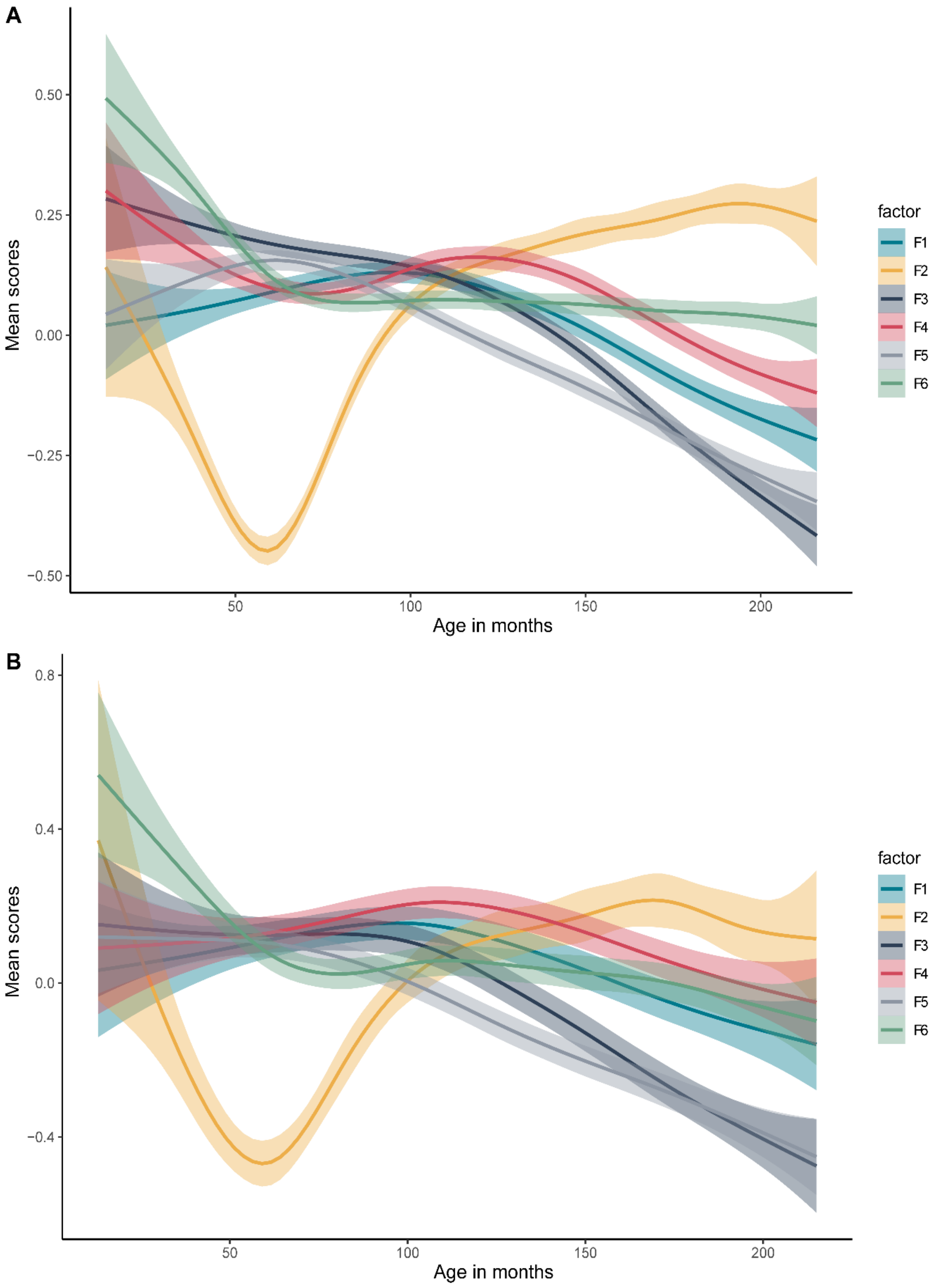

A. Age related trajectories in males. B Age related trajectories in females. The six factors are: 1. Insistence on sameness (F1); 2. Social interaction (F2); 3. Sensory-motor behaviour (F3); 4. Self-injurious behaviour (F4); 5. Idiosyncratic repetitive speech and behaviour (F5); 6. Communication skills (F6). F2 primarily consists of items related to Social interaction at ages 4 – 5, hence the trajectory likely reflects recall bias in participants. Shaded region indicates 95% confidence intervals for the loess curves.

Supplementary Figure 5: Effect directions for the significant PGS associations

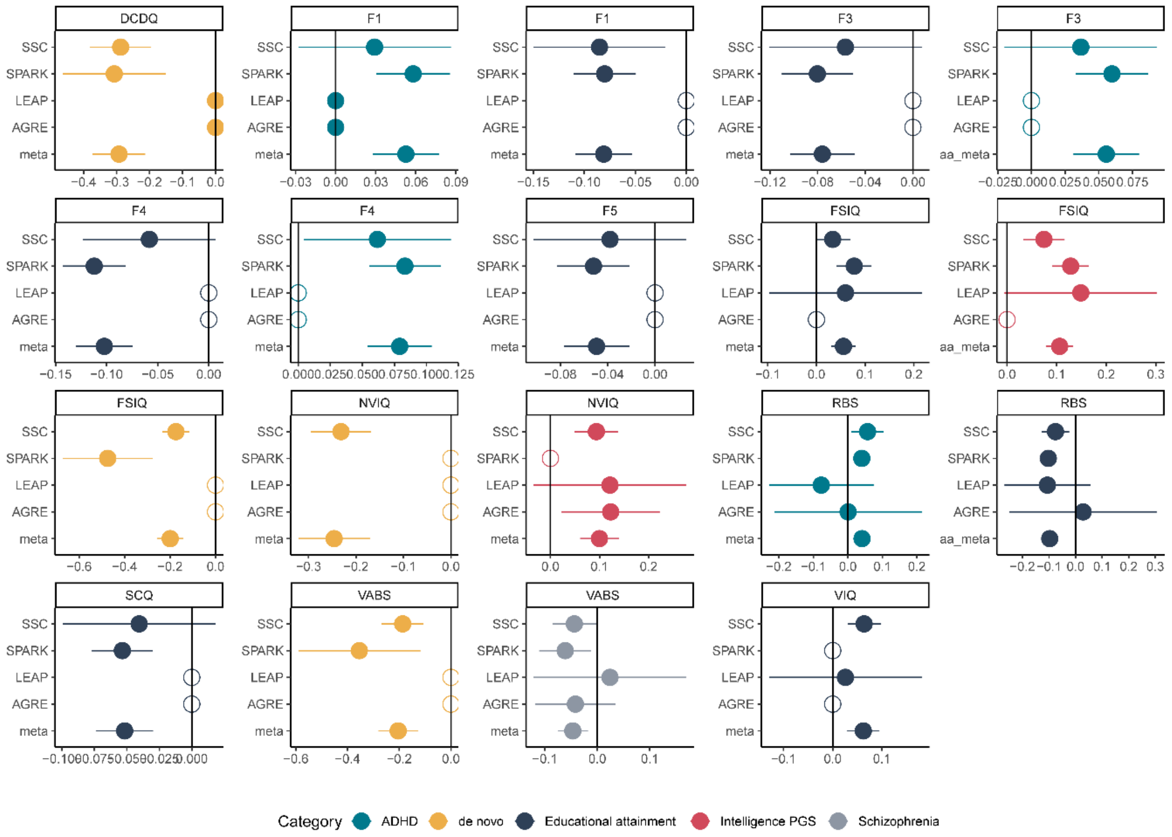

Regression beta (centre point) and 95% confidence intervals for the significant PGS-feature associations by cohort. Empty circles represent cohorts where the phenotypes were not available (F1, F2, F3, F4, F5, and SCQ were not available in AGRE and LEAP; full-scale IQ and verbal IQ were not available in AGRE, non-verbal IQ and verbal IQ were not available in SPARK). Meta represents the meta-analysed estimates and associated 95% confidence intervals. Adaptive behaviour was measured using the composite scores from the Vinelands Adaptive Behaviour Scales. The five factors are: 1. Insistence on sameness (F1); 2. Social interaction (F2); 3. Sensory-motor behaviour (F3); 4. Self-injurious behaviour (F4); 5. idiosyncratic repetitive speech and behaviour (F5). Sample sizes are provided in Supplementary Table 8.

Supplementary Figure 6: Differences in autism PGS by sex, and diagnostic and carrier status

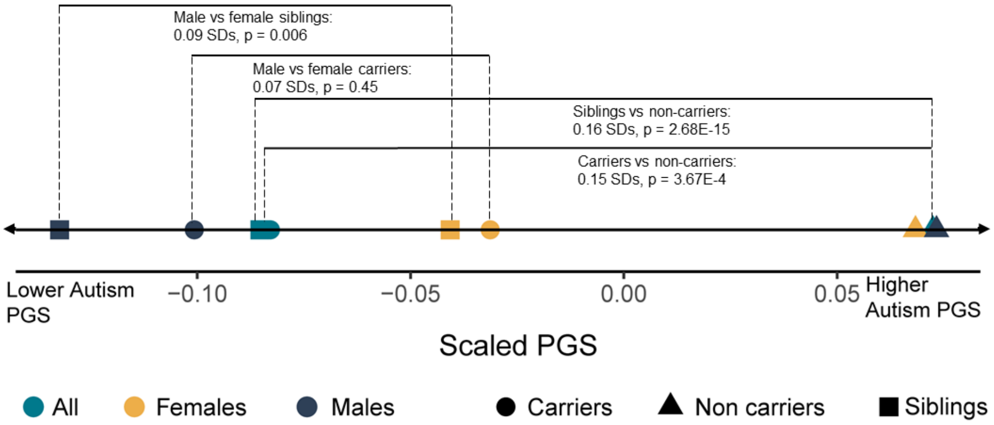

Sex	Category	Mean	N
All	Carriers	-0.082	579
All	Non carriers	0.072	4997
All	Siblings	-0.085	3681
Females	Carriers	-0.031	149
Females	Non carriers	0.068	868
Females	Siblings	-0.041	1888
Males	Carriers	-0.101	430
Males	Non carriers	0.073	4129
Males	Siblings	-0.132	1793

Differences in standardised autism PGS (mean = 0, standard deviation = 1). Line is drawn to scale. Standard deviations (SDs) and p-values have been provided for select comparisons where visual inspection of the plot identified sizable differences in PGS between groups. p-values have been calculated using linear regression using autism PGS residualised for 10 genetic principal components, and with sex (non-stratified comparisons only) and cohort included as covariates. The table provides the mean PGS and sample size for each group.

Supplementary Figure 7: Leave-one-out analyses for genotype-developmental disability associations

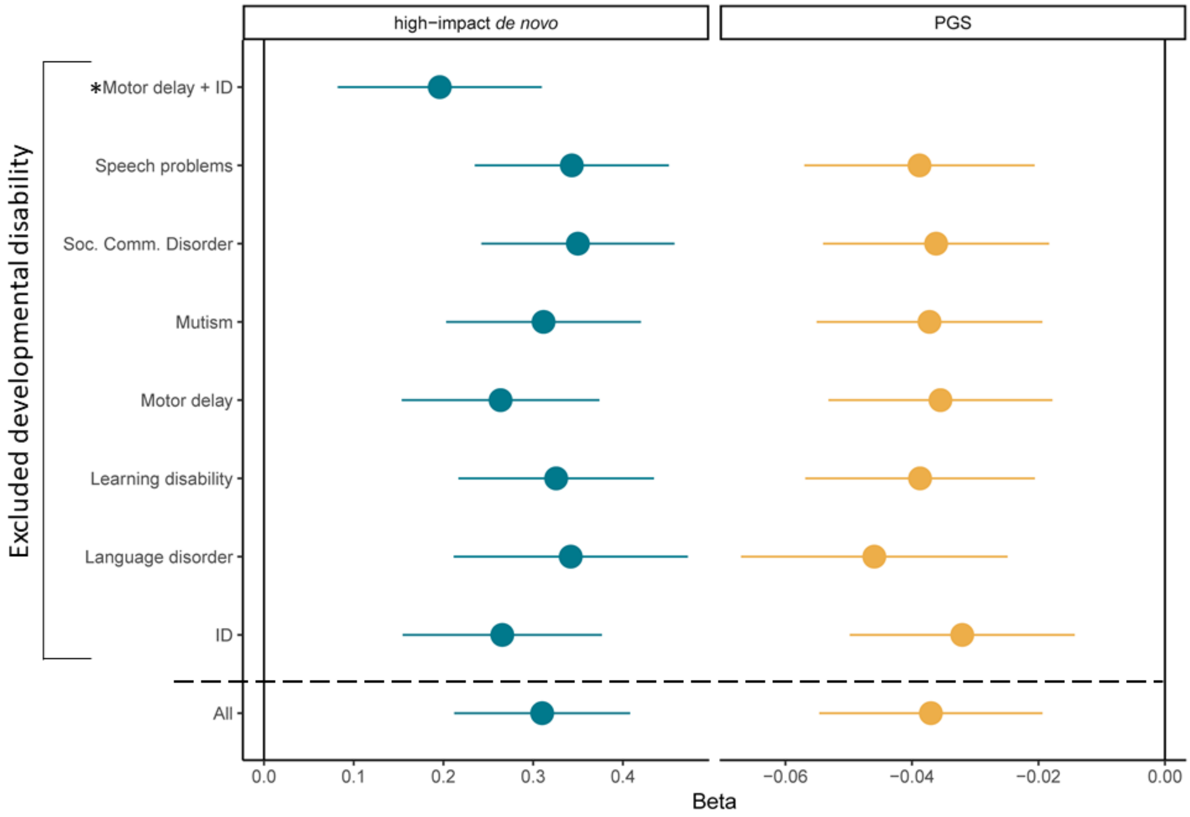

*Leave-one-out analyses for the associations between autism polygenic scores (N = 13,435) or high-impact de novo variants (N = 3,089) and count of developmental disabilities. Regression betas (centre point) and 95% confidence intervals provided. For high-impact de novo variants, we additionally conducted leave-one-out analyses after excluding both motor delay and ID (indicated using *), given the associations between high-impact variants and both IQ and motor coordination. We also provide the beta and 95% confidence intervals for the original regression for the count of all seven developmental disabilities ('All') for comparison.*

Supplementary Figure 8: Distribution of full-scale IQ bins in SPARK and SSC combined

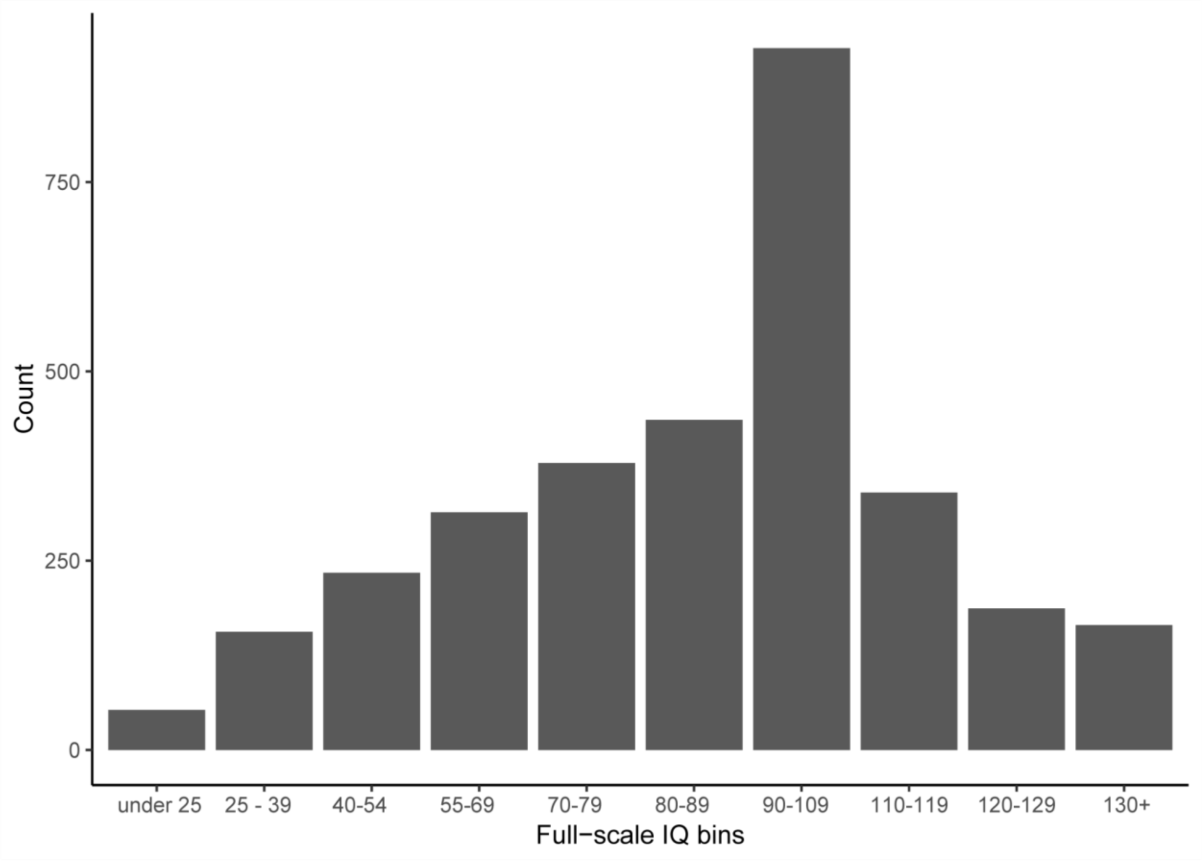

Frequency histogram of binned full-scale IQ scores from the SPARK and SSC cohorts.

Supplementary Figure 9: Distribution of individuals of European ancestries in SPARK, SSC, and ABCD by genetic principal components

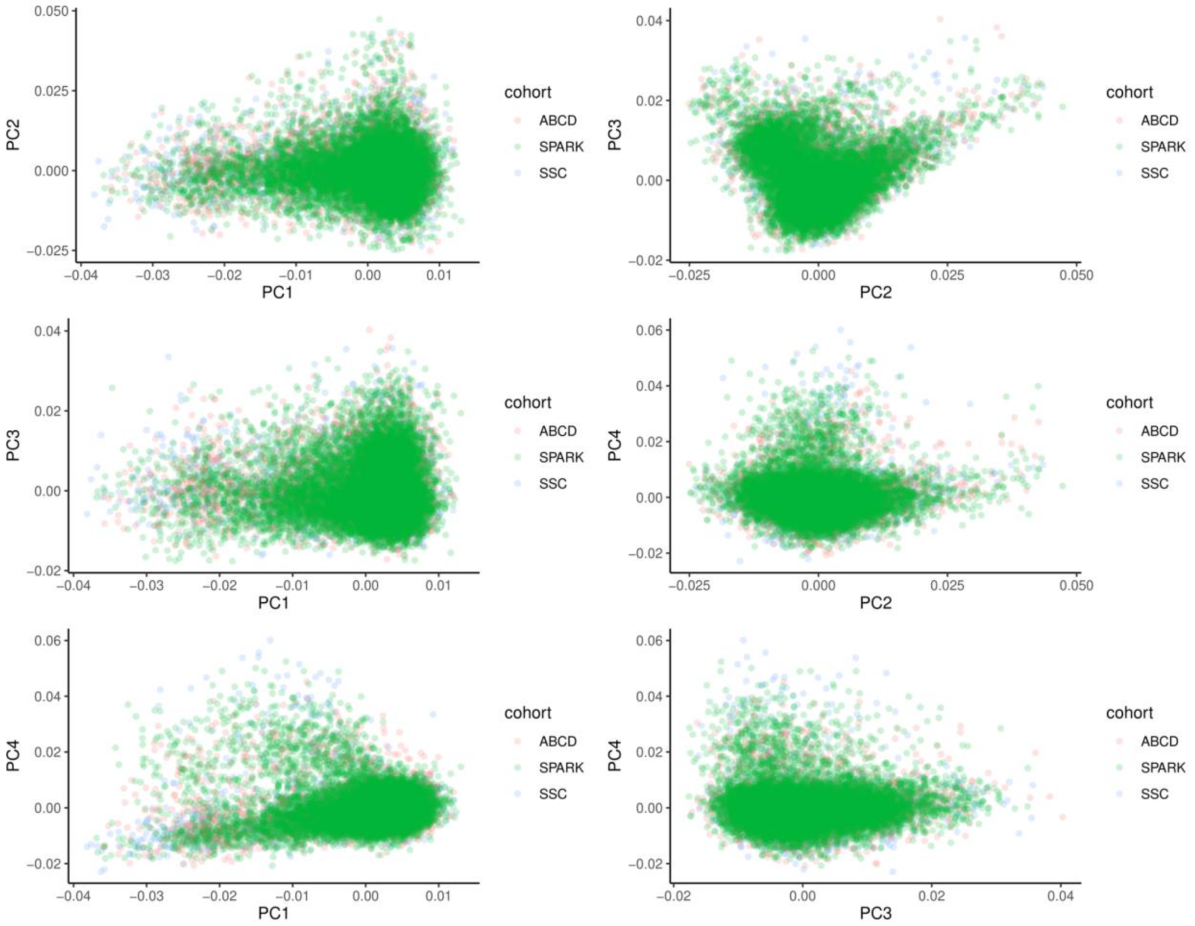

Individuals of predominantly European ancestries from the SPARK, ABCD, and SSC cohorts plotted based on the first four genetic principal components. Visual inspection of the plots identified substantial alignment between the three cohorts in the principal component space.
