# Supplementary material for: Genetic correlates of phenotypic heterogeneity in autism
Source: Nat Genet. 2022 Jun 2;54(9):1293–304. doi: 10.1038/s41588-022-01072-5 (PMC9470531; doi:10.1038/s41588-022-01072-5)
Supplement: Supplementary file 2 — Reporting Summary. [file 41588_2022_1072_MOESM2_ESM.pdf]

nature portfolio

Corresponding author(s): Varun Warrier

Last updated by author(s): Feb 14, 2022

Reporting Summary

Nature Portfolio wishes to improve the reproducibility of the work that we publish. This form provides structure for consistency and transparency in reporting. For further information on Nature Portfolio policies, see our Editorial Policies and the Editorial Policy Checklist.

Statistics

For all statistical analyses, confirm that the following items are present in the figure legend, table legend, main text, or Methods section.

n/a	Confirmed	
☐	☒	The exact sample size (n) for each experimental group/condition, given as a discrete number and unit of measurement
☐	☒	A statement on whether measurements were taken from distinct samples or whether the same sample was measured repeatedly
☐	☒	The statistical test(s) used AND whether they are one- or two-sided<br>*Only common tests should be described solely by name; describe more complex techniques in the Methods section.*
☐	☒	A description of all covariates tested
☐	☒	A description of any assumptions or corrections, such as tests of normality and adjustment for multiple comparisons
☐	☒	A full description of the statistical parameters including central tendency (e.g. means) or other basic estimates (e.g. regression coefficient) AND variation (e.g. standard deviation) or associated estimates of uncertainty (e.g. confidence intervals)
☐	☒	For null hypothesis testing, the test statistic (e.g. F, t, r) with confidence intervals, effect sizes, degrees of freedom and P value noted<br>*Give P values as exact values whenever suitable.*
☒	☐	For Bayesian analysis, information on the choice of priors and Markov chain Monte Carlo settings
☒	☐	For hierarchical and complex designs, identification of the appropriate level for tests and full reporting of outcomes
☐	☒	Estimates of effect sizes (e.g. Cohen's d, Pearson's r), indicating how they were calculated

Our web collection on statistics for biologists contains articles on many of the points above.

Software and code

Policy information about availability of computer code

Data collection	No software has been used for data collection

Data analysis	● Genetic QC and imputation in SSC: vwarrier/SSC_liftover_imputation: Basic scripts used for imputing the SSC genotyped datasets (github.com)<br>● Genetic QC and imputation in SPARK: vwarrier/SPARK_QC_imputation: QC and imputation of the SPARK dataset (github.com)<br>● Genetic QC and imputation in ABCD: vwarrier/ABCD_geneticQC (github.com)<br>● Bespoke genetic analyses: vwarrier/autism_heterogeneity: This git has the code for the heterogeneity in autism project (github.com)<br>We used the following software packages:<br>● PRScs: getian107/PRScs: Polygenic prediction via continuous shrinkage priors (github.com); March 4, 2021 version<br>● TOPMED imputation server: TOPMed Imputation Server (nih.gov)<br>● Plink: PLINK 2.0 (cog-genomics.org)<br>● GCTA-GREML: GCTA v 1.93.3 beta(cnsgenomics.com)<br>● PCGC: PCGC Regression | dougspeed.com; LDAK 5.1 linux<br><br>R packages:<br>1. Psych 2.1.6<br>2. Cocor 1.1-3<br>3. Lavaan 0.6-5<br>4. MASS 7.3-54<br>5. lme4 1.1-27.1

For manuscripts utilizing custom algorithms or software that are central to the research but not yet described in published literature, software must be made available to editors and reviewers. We strongly encourage code deposition in a community repository (e.g. GitHub). See the Nature Portfolio guidelines for submitting code & software for further information.

Data

Policy information about availability of data

All manuscripts must include a data availability statement. This statement should provide the following information, where applicable:

- Accession codes, unique identifiers, or web links for publicly available datasets
- A description of any restrictions on data availability
- For clinical datasets or third party data, please ensure that the statement adheres to our policy

Genetic and phenotypic data for SFARI and SPARK are available upon application and approval from the Simons Foundation (SFARI | Autism Cohorts). Approved researchers can obtain the SPARK and SSC population dataset described in this study by applying at https://base.sfari.org. Data for AGRE is available upon application and approval from Autism Speaks (AGRE - Autism Genetic Resource Exchange | Autism Speaks). Data for EU-AIMS Leap is available upon application and approval to the EU-AIMS LEAP committee (The LEAP Study (eu-aims.eu)). Ddg2p phenotype can be obtained here: DECIPHER v11.9: Mapping the clinical genome (deciphergenomics.org). GWAS data availability: Hair colour (https://atlas.ctglab.nl/traitDB/3495); Schizophrenia and ADHD (Download Results | Psychiatric Genomics Consortium (unc.edu)); intelligence (GWAS Summary Statistics | CTG (cncr.nl)); educational attainment (SSGAC Login (thessgac.com)).

Field-specific reporting

Please select the one below that is the best fit for your research. If you are not sure, read the appropriate sections before making your selection.

☒ Life sciences ☐ Behavioural & social sciences ☐ Ecological, evolutionary & environmental sciences

For a reference copy of the document with all sections, see nature.com/documents/nr-reporting-summary-flat.pdf

Life sciences study design

All studies must disclose on these points even when the disclosure is negative.

Sample size	We used the largest available sample size, combining data from four different cohorts.
Data exclusions	We excluded individuals who did not pass genetic quality control.
Replication	No direct replication was conducted. We conducted meta-analyses of all available data, and used orthogonal methods to validate the results.
Randomization	Randomization was only needed for factor analyses. This was done in R. No randomization was needed for other analyses. For association analyses using polygenic scores and SNP heritability analyses we included sex, age and genetic principal components.
Blinding	No blinding was conducted in this study as autistic individuals and non-autistic individuals were identified by diagnosis.

Reporting for specific materials, systems and methods

We require information from authors about some types of materials, experimental systems and methods used in many studies. Here, indicate whether each material, system or method listed is relevant to your study. If you are not sure if a list item applies to your research, read the appropriate section before selecting a response.

Materials & experimental systems

n/a	Involved in the study
☒	☐ Antibodies
☒	☐ Eukaryotic cell lines
☒	☐ Palaeontology and archaeology
☒	☐ Animals and other organisms
☐	☒ Human research participants
☒	☐ Clinical data
☒	☐ Dual use research of concern

Methods

n/a	Involved in the study
☒	☐ ChIP-seq
☒	☐ Flow cytometry
☒	☐ MRI-based neuroimaging

Human research participants

Policy information about studies involving human research participants

Population characteristics	For factor analyses, we restricted our analyses to autistic individuals from the Simons Simplex Collection (SSC) and SPARK cohorts. Participants had to have completed the two phenotypic measures (details below) to be included in the factor analyses. We also excluded autistic individuals with incomplete entries in either of the two measures (N = 5,754 only in SPARK). This resulted in 1,803 participants (N = 1,554 males) in SSC, 14,346 (N = 11,440 males) in SPARK version 3 and 8,271 (N = 6,262 males) in extra entries from SPARK version 5 (SSC: Mean age = 108.75, SD = 43.29 ; SPARK version 3: Mean age = 112.11 months, SD = 46.43; SPARK version 5: Mean age = 111.22 months, SD = 48.19). Only the SCQ was available for siblings in SPARK.<br>We conducted analyses using data from four cohorts of autistic individuals: The Simons Simplex Collection (SSC, N = 8,813)30, the Autism Genetic Resource Exchange (AGRE, CHOP sample) (Nmax = 1,200)64, the AIMS-2-TRIALS LEAP sample (Nmax = 262)65, and SPARK (N = 29,782)31. For sibling comparisons, we included siblings from SSC (N = 1,829) and SPARK (N = 12,260). For trio-based analyses, we restricted to complete trios in SSC (N = 2,234) and SPARK (N = 4,747). For all analyses we restricted the sample to autistic individuals who passed genetic quality control and who had phenotypic information.
Recruitment	Participants were recruited through clinics, labs, and online. This was done by others.
Ethics oversight	University of Cambridge's Human Biology Research Ethics Committee provided ethical approval to analyse pseudonymised phenotypic and genetic data.

Note that full information on the approval of the study protocol must also be provided in the manuscript.
